# Disruption of the kringle 1 domain of prothrombin leads to late onset mortality in zebrafish

**DOI:** 10.1038/s41598-020-60840-7

**Published:** 2020-03-04

**Authors:** Steven J. Grzegorski, Zhilian Hu, Yang Liu, Xinge Yu, Allison C. Ferguson, Hasam Madarati, Alexander P. Friedmann, Deepak Reyon, Paul Y. Kim, Colin A. Kretz, J. Keith Joung, Jordan A. Shavit

**Affiliations:** 1 Department of Pediatrics, University of Michigan, Ann Arbor, MI USA; 2 Department of Medicine, McMaster University, Hamilton, ON Canada; 3 Thromosis and Atherosclerosis Research Institute, Hamilton, ON Canada; 4 Molecular Pathology Unit, Massachusetts General Hospital, Charlestown, MA USA; 5 Department of Pathology, Harvard Medical School, Boston, MA USA; 6 Present Address: Oxford University, Oxford, UK; 7 Present Address: Molecular Innovations, Inc., Novi, MI USA; 8 Present Address: Editas Medicine Inc., Cambridge, MA USA

**Keywords:** Haematological diseases, Mechanisms of disease

## Abstract

The ability to prevent blood loss in response to injury is a conserved function of all vertebrates. Complete deficiency of the central clotting enzyme prothrombin has never been observed in humans and is incompatible with postnatal life in mice, thus limiting the ability to study its role *in vivo*. *Z*ebrafish are able to tolerate severe hemostatic deficiencies that are lethal in mammals. We have generated a targeted genetic deletion in the kringle 1 domain of zebrafish prothrombin. Homozygous mutant embryos develop normally into the mid-juvenile stage but demonstrate complete mortality by 2 months of age primarily due to internal hemorrhage. Mutants are unable to form occlusive venous and arterial thrombi in response to endothelial injury, a defect that was phenocopied using direct oral anticoagulants. Human prothrombin engineered with the equivalent mutation exhibits a severe reduction in secretion, thrombin generation, and fibrinogen cleavage. Together, these data demonstrate the conserved function of thrombin in zebrafish and provide insight into the role of kringle 1 in prothrombin maturation and activity. Understanding how zebrafish are able to develop normally and survive into early adulthood without thrombin activity will provide important insight into its pleiotropic functions as well as the management of patients with bleeding disorders.

## Introduction

Maintaining blood flow in a closed circulatory system requires a delicate balance between pro- and anticoagulant factors. Disequilibrium of these factors in either direction can lead to pathology. In response to vascular injury, the balance shifts towards coagulation in an effort to stabilize blood clots and prevent exsanguination. Critical to this clot stabilization is the activation of prothrombin, a vitamin K-dependent clotting factor, to form the central clotting enzyme, thrombin^1^. Thrombin cleaves soluble fibrinogen into fibrin monomers, which then polymerize to form the insoluble fibrin clot^2^. Additionally, thrombin interacts with protease activated receptors (PARs) on the surface of various cells, including platelets, which makes it the most potent endogenous agonist of primary hemostasis^3^. Human prothrombin (F2) variants have been linked to both thrombophilia and bleeding diatheses. The most common variant (~2% in European populations)^4^ is a guanine to adenine transition in the 3’ untranslated region at position 20210 that leads to increased plasma prothrombin levels and a 2–3 fold elevated risk of deep vein thrombosis^5,6^. Congenital prothrombin deficiencies result in a bleeding diathesis, but are rare. Acquired deficiencies due to liver failure or vitamin K deficiency are more common^7,8^.

Structurally, prothrombin consists of six domains: signal peptide, propeptide, Gla, kringle 1, kringle 2, and serine protease; a domain composed of a light and heavy chain^1^. Following translation, the propeptide targets the protein for post-translational gamma-carboxylation of the glutamic acid residues within the Gla domain. This vitamin K-dependent process is necessary for proper localization of the mature zymogen to the activated membrane surface^9^. While relatively understudied, the kringle domains are thought to interact with activated factor V (FVa) during assembly of the prothrombinase complex (FVa and activated factor X (FXa))^10–12^. Cleavage of prothrombin by the prothrombinase complex results in fragment 1.2 (the Gla and 2 kringle domains) and thrombin (light and heavy chains).

Targeted mutagenesis of *F2* in mice demonstrates 50% mortality by embryonic day 10.5 with hemorrhage associated with suspected defects in yolk sac vasculature. Mutants surviving to birth succumb to hemorrhage by postnatal day 1^13,14^. Loss of other common pathway factors, including FV and FX, show a similar pattern of bimodal lethality^15,16^. These data are suggestive of a secondary role beyond the coagulation system during development. More recent studies reveal that conditional knockout of prothrombin in adult mice leads to mortality due to hemorrhagic events within 5–7 days, although there remains a residual level of prothrombin^17^. Combined with the long half-life of prothrombin (60–72 hours)^18^, it is likely that the adult mice do not achieve complete deficiency prior to lethality.

Due to early lethality of complete genetic knockouts of prothrombin in mice, the nuances of thrombin’s role in *in vivo* hemostasis and development are difficult to study. Zebrafish (*Danio rerio*) has advantages for the investigation of early development because of its high fecundity, external fertilization, and transparent development. Embryonic and adult studies have demonstrated the benefits of zebrafish for the study of hemostatic and other human diseases^19,20^. Conservation of coagulation factors in zebrafish is well characterized with a variety of techniques and genetic models^21–27^. FX knockout (*f10*^−/−^) zebrafish survive several months into adulthood before succumbing to lethal hemorrhage, suggesting that the model is more resistant to extreme disturbances in hemostasis than mammals^28^. Notably, evaluation of *f10*^−/−^ fish vasculature shows no abnormalities in the embryonic period.

Complete prothrombin deficiency has never been seen in humans and is incompatible with life in mice, limiting the ability to understand the entirety of prothrombin’s *in vivo* functions. Therefore, we have now used genome editing TALENs (transcription activator-like effector nucleases) to produce a genetic knockout of the prothrombin gene (*f2*) in zebrafish. We show here that loss of prothrombin in zebrafish does not result in severe developmental defects but does prevent the formation of clots in response to endothelial injury and leads to early mortality at 2–3 months of age. Furthermore, the mutation generated lends insight into the role of the kringle 1 domain in prothrombin biosynthesis and activation.

## Results

### Zebrafish prothrombin demonstrates a high degree of sequence conservation

**Figure 1:**
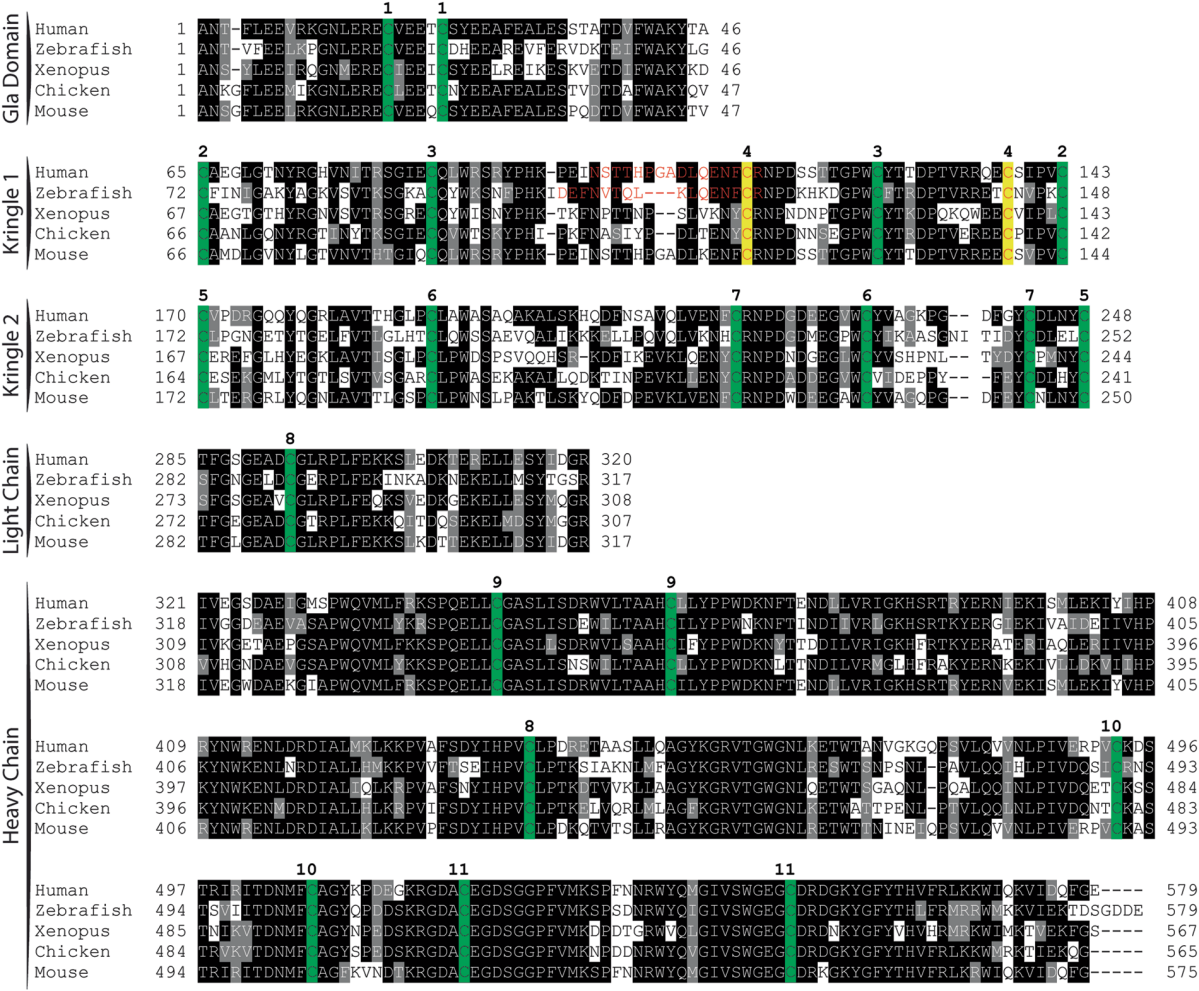
Peptide sequence alignment shows strong conservation of prothrombin across a broad range of species. Sequences shown include the conserved domains. Numbering begins at the first residue after the propetide according to the prothrombin numbering scheme. Sequences are shaded to indicate degree of conservation. Green colored residues indicate conserved cysteines. Red colored residues represent amino acids altered by mutagenesis (Δ15; C138A) with the affected paired cysteines highlighted in yellow. Numbers above alignment indicate paired cysteines in human prothrombin.

Mature zebrafish prothrombin is a 579 amino acid protein that shares 55% identity and 72% homology with human prothrombin, including 68% identity and 86% homology when comparing the regions corresponding to thrombin. Zebrafish prothrombin is detected within the first day of development and leads to measurable fibrin forming activity by 48 hours^29^. Overall, the prothrombin sequence is highly conserved across vertebrates, with complete conservation of cysteine residues, suggesting the conservation of major structural features such as the kringle domains (Fig. 1).

### Zebrafish thrombin activity is inhibited by direct oral anticoagulants

**Figure 2:**
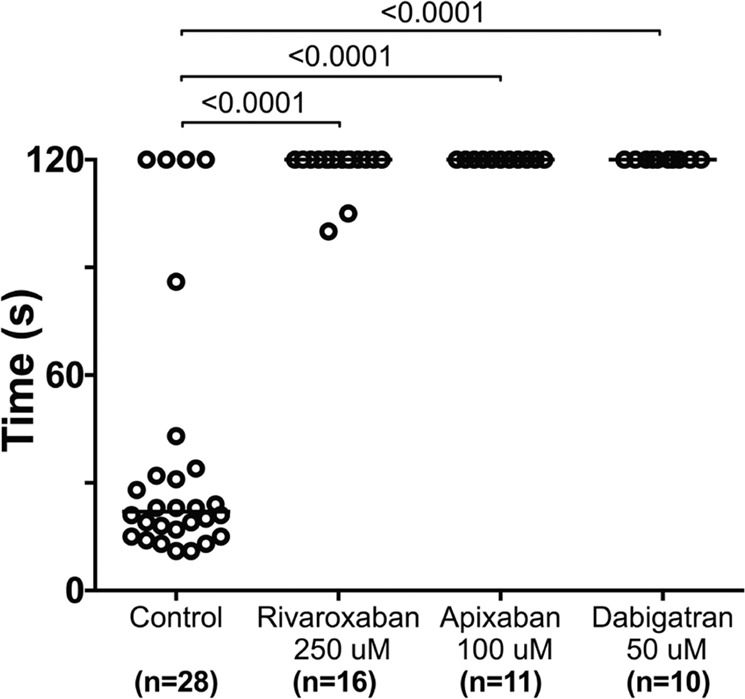
Direct oral anticoagulants prevent occlusive thrombus formation in zebrafish. 5 dpf larvae were exposed to chemical inhibitors of thrombin (dabigatran) and FXa (apixaban, rivaroxaban) for 24 hours. This resulted in the inability to form occlusive venous thrombi at 6 dpf in wild-type larvae following laser-mediated endothelial injury.

To test the relative conservation between zebrafish and human thrombin, larvae were treated with three direct oral anticoagulants: direct thrombin inhibitor (dabigatran) as well as direct FXa inhibitors (rivaroxaban and apixaban). Following 24 hours of treatment, 6 dpf larvae exposed to dabigatran etexilate, rivaroxaban, or apixaban were unable to form occlusive thrombi in response to injury (Fig. 2). These data show that the carboxylesterase function required to convert dabigatran etexilate to dabigatran is conserved in zebrafish, as is the ability of dabigatran to inhibit zebrafish thrombin. Apixaban and rivaroxaban also demonstrate conserved inhibition of FXa, which inhibits prothrombin activation. In humans, mean plasma concentrations of all 3 inhibitors are in the range of 100–600 nM^30,31^. Similar concentrations of dabigatran and 10–100 fold higher concentrations of rivaroxaban have been shown to impair clot formation following tail bleeds in mice^32,33^. Although difficult to predict, the low polarity of the 3 molecules leads us to estimate that the molecules will be poorly absorbed from the media and result in similar tissue levels to those seen in humans^34^.

### Targeted genome editing induces a deletion resulting in decreased *f2* expression

**Figure 3:**
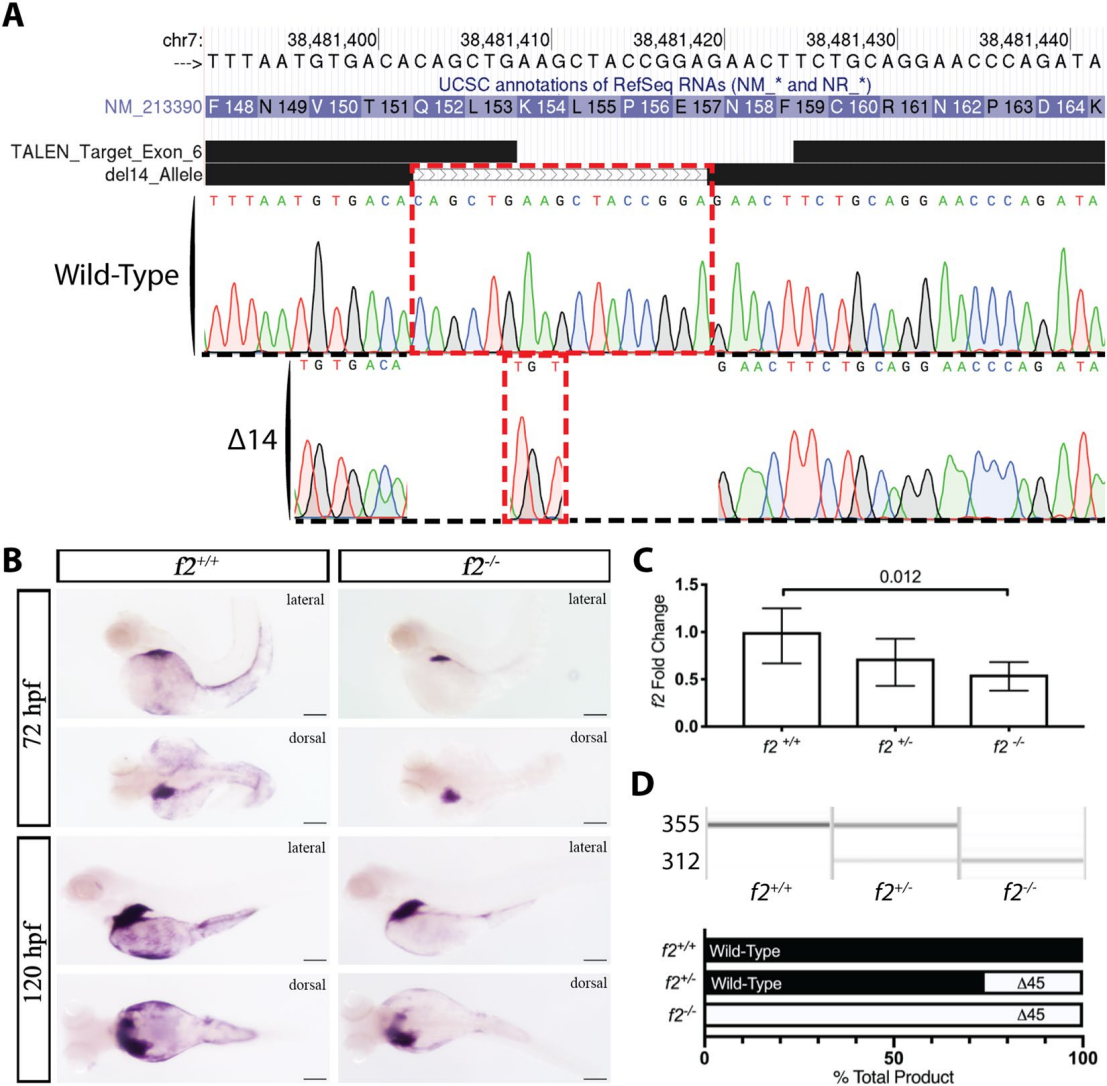
Genome editing creates a 14 bp genomic deletion with a resulting decrease in mRNA expression. (**A**) Alignment of Sanger sequencing with the chromosome 7 genomic region showed an overall 17 bp genomic deletion replaced with a 3 bp insertion; outlined in red, resulting in a net 14 bp deletion. (**B**) *in situ* hybridization demonstrated reduction of transcript at 72 and 120 hours post fertilization in homozygous mutants compared to control siblings. Spatial regulation remained intact with expression restricted primarily to the liver. (**C**) qPCR data of *f2* expression reveals significant decrease of 45% in the homozygous mutant embryos. (**D**) Semi-quantitative RT-PCR of embryos shows a mutant band ~30 bp smaller than expected (later shown to be a 45 bp deletion, Fig. 4). Quantitation of the bands reveal that the mutant band is only 26% of the total in heterozygotes.

Having established the functional conservation of prothrombin, we sought to analyze the long-term effects of thrombin deficiency using a genetic model. Utilizing TALEN-mediated genome editing, exon 6 of *f2* was targeted with the aim of creating a frameshift and subsequent nonsense mutation prior to the protease domain. Sequencing data showed a 14 bp deletion within the genomic region homologous to the human prothrombin kringle 1 domain (Fig. 3A). *In situ* hybridization demonstrated decreased, but not absent *f2* mRNA in homozygous mutants at 3 and 5 dpf compared to wild-type siblings (Fig. 3B). This is further supported by quantitative RT-PCR data demonstrating a 45% reduction in mRNA transcript in homozygous mutants (Fig. 3C). To characterize the residual mutant transcript, semi-quantitative RT-PCR was performed using primers flanking the mutation site. Only wild-type and mutant bands were seen in pools of wild-type and homozygous mutant embryos, respectively. In heterozygous embryos, the computed molar amount of the mutant band was only 26% of the total, with the remainder being wild-type (Fig. 3D). Notably, the homozygous mutant transcript was roughly 30 base pairs smaller than expected.

### Genomic deletion reveals a cryptic splice site that creates an alternative splice variant

**Figure 4:**
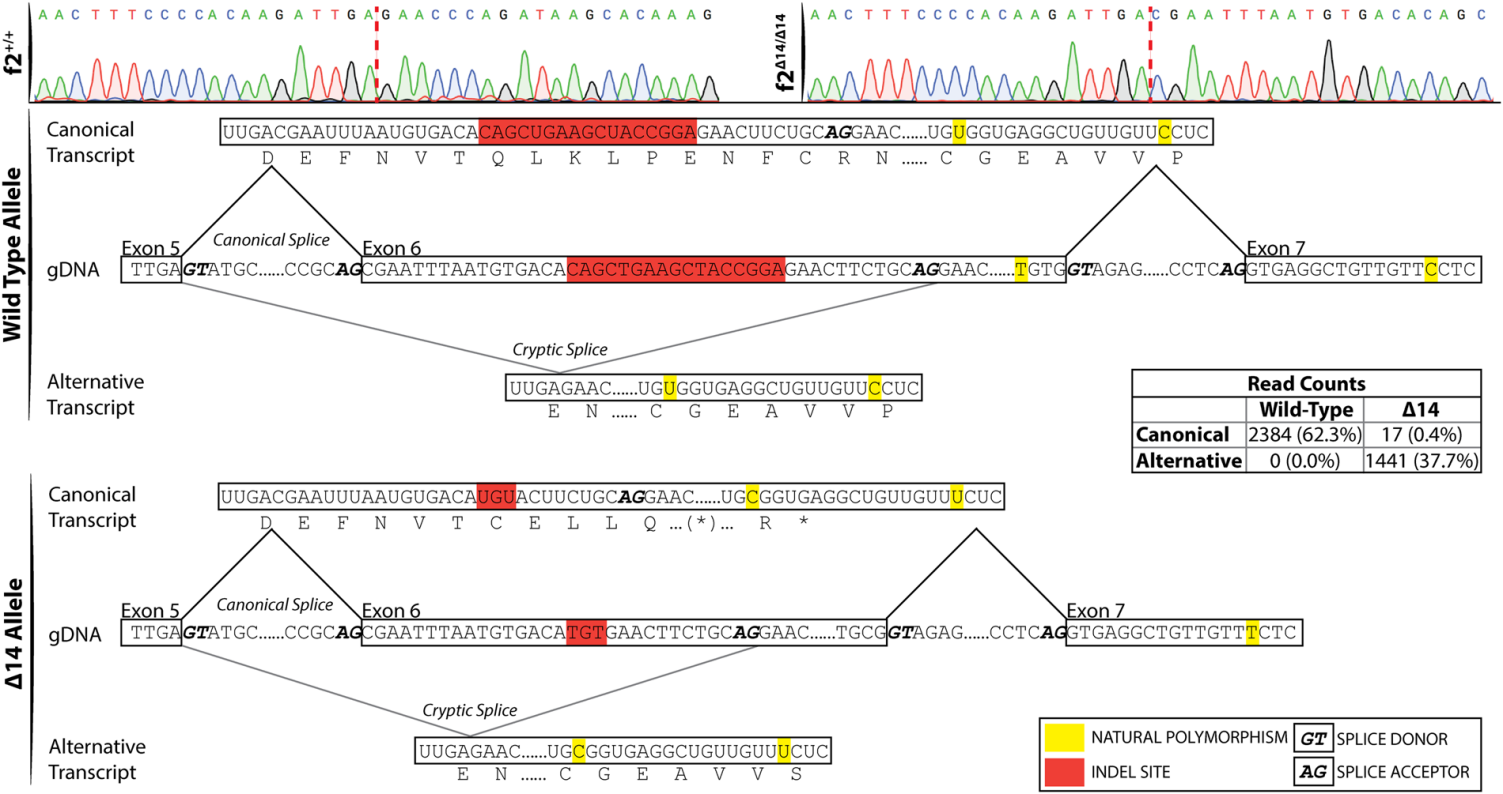
Single molecule real time sequencing of *f2*^+/Δ14^ mRNA demonstrates altered splicing in *f2* following a deletion in exon 6. (top) Sanger sequencing of *f2*^+/+^ and *f2* ^Δ14/Δ14^ cDNA reveals a canonical splice donor but alternative splice acceptor (red dashed lines indicate splice junctions). Allelic SNPs in exon 7 allowed the sorting of transcripts by their genomic allele in mRNA extracted from heterozygotes. Wild-type transcripts (middle) solely demonstrated canonical splicing. Mutant transcripts (bottom) primarily exhibited alternative splicing to a cryptic splice site with a resulting 45 bp inframe deletion, as well as low frequency canonical splicing with a resulting 14 bp out of frame deletion. gDNA, genomic DNA.

To identify potential splice variants, full length *f2* cDNA was sequenced from *f2*^+/+^ and *f2*^Δ14/Δ14^ larvae (Fig. 4, top). This revealed that there is a cryptic splice acceptor 3′ to the 14 bp genomic deletion in exon 6. In contrast to the canonical splicing which forms a transcript containing the 14 bp deletion, alternative splicing from the exon 5 splice donor site to the cryptic splice site generates a 45 bp deletion in kringle 1 of prothrombin (Fig. 4). Notably, this maintains the reading frame and replaces DEFNVTQLKLQENFCR with a single glutamic acid for a net loss of 15 amino acids. This deletion includes a highly conserved cysteine (C119 in the zebrafish peptide) predicted by homology to form a disulfide bond with zebrafish C143 (Fig. 1). The protein product of this alternatively spliced transcript is referred to as Δ15. To understand the distribution of splicing, we performed deep SMRT sequencing of *f2* cDNA from *f2*^+/Δ14^ mutant larvae at 3 dpf. Overall, 3842 consensus sequences were analyzed. Using downstream allelic polymorphisms, the reads were sorted into wild-type and *f2*^Δ14^ haplotypes. Of the 2384 reads derived from the wild-type locus, 100% underwent canonical splicing (Fig. 4, middle). In contrast, only 17/1458 (1.2%) mutant locus reads displayed canonical splicing (Fig. 4, bottom) and the remainder made use of the cryptic splice site to form the Δ15 transcript (1441/1458, 98.8%).

### Disruption of the prothrombin kringle 1 domain results in adult lethality due to internal hemorrhage

**Figure 5:**
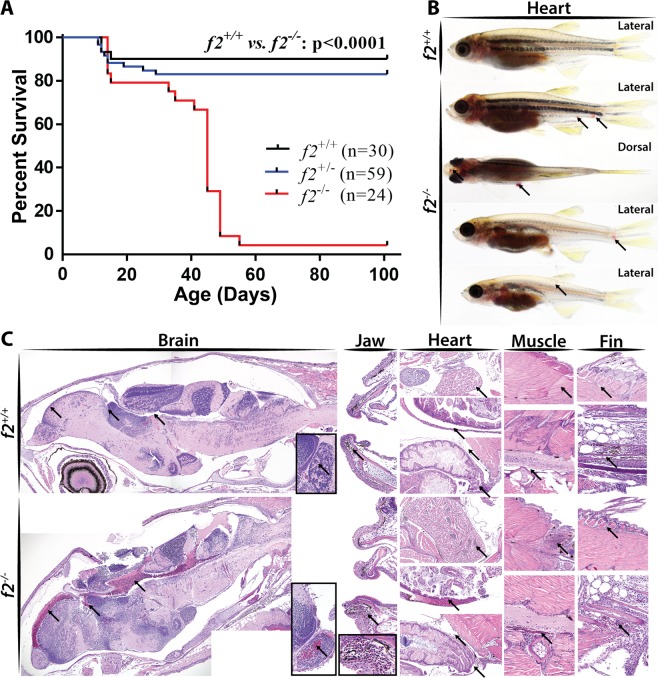
Loss of prothrombin results in early lethality due to hemorrhage. (**A**) Survival curve demonstrating significant mortality by 2 months of age in *f2*^−/−^ siblings, log-rank (Mantel-Cox) analysis. (**B**) Examples of grossly visible intracranial, intramuscular, and fin bleeds (arrows). (**C**) Histological sections of wild-type and *f2*^−/−^ siblings demonstrated microscopic bleeds in the brain, jaw, heart, muscle, and fins. Arrows point to pools of erythrocytes and unaffected comparable tissue in the control.

To examine the functional consequence of kringle 1 disruption, we evaluated homozygous mutant fish at various stages of development. Beginning around 1 month of age, *f2*^Δ14/Δ14^ fish showed signs of overt internal hemorrhage and the majority (23/24) died by adulthood, defined as 90 days of age (Fig. 5A,B). Gross intracranial and fin hemorrhage was visible, and histological analysis demonstrated the occurrence of bleeds within the head, jaw, muscle, fins, and around the heart and abdomen (Fig. 5C).

### Homozygous mutant larvae fail to form thrombi in response to endothelial injury

**Figure 6:**
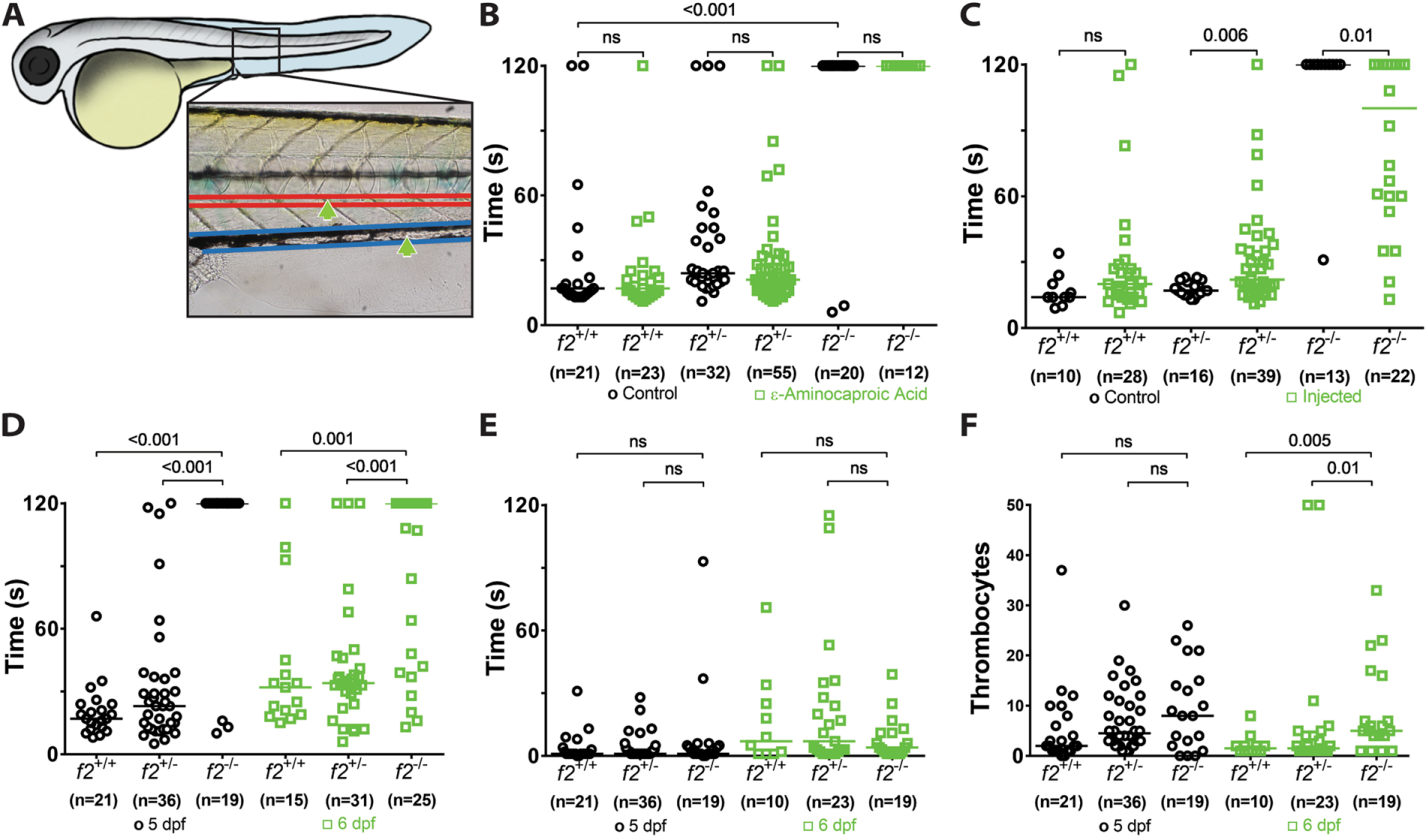
Loss of thrombin activity results in defects in secondary hemostasis. (**A**) Larvae were immobilized in agarose, subjected to laser-mediated endothelial injury (green arrow) of the venous (PCV, blue) or arterial (dorsal aorta, red) circulation, and followed for 2 minutes by a blinded observer. (**B**) Genetic ablation of *f2* resulted in the inability to form induced PCV thrombi at 3 dpf and was not influenced by inhibiting fibrinolysis (ɛ-aminocaproic acid treatment, blue). (**C**) Overexpression of human *F2* cDNA (blue) rescued the ability to form thrombi in the PCV at 3 dpf. (**D**) Homozygous mutant larvae demonstrated a significant impairment in arterial thrombus formation at 5 and 6 dpf without any changes in the time to initial thrombocyte attachment (**E**). (**F**) The number of thrombocytes attached to the site of injury in 2 minutes was significantly increased at 6 dpf in *f2* homozygous mutants. Statistical significance assessed by Mann-Whitney *U* testing.

Given that 80% of *f2*^Δ14/Δ14^ fish were able to survive development to the juvenile stage (30–90 days of age), we assessed the possibility that there could be residual detectable thrombin activity in early embryos and larvae. Thrombin has roles in both primary and secondary hemostasis, platelet activation and fibrin formation, respectively. These roles were evaluated using laser-mediated endothelial injury models of arterial and venous thrombus formation (Fig. 6A). Homozygous mutant larvae were unable to form occlusive thrombi in the venous system in response to injury. This was refractory to treatment with the fibrinolytic inhibitor ε-aminocaproic acid, further confirming a lack of fibrin generation (Fig. 6B). To confirm that defects in hemostasis were not due to off target effects of genome editing, embryos were injected at the one-cell stage with plasmid expressing human *F2* cDNA driven by the constitutively active cytomegalovirus promoter^35,36^. Endothelial injury at 3 dpf induced clot formation within 2 minutes in 50% of homozygous embryos in contrast to uninjected controls (Fig. 6C). To assess the role of thrombin in the zebrafish arterial system, the *f2*^Δ14^ mutation was bred into the *Tg(cd41-egfp)* background in which circulating thrombocytes express GFP. At 5 and 6 dpf, *f2*^Δ14/Δ14^ larvae had a decreased ability to form occlusive arterial thrombi (Fig. 6D). The time to thrombocyte attachment was not statistically different between groups (Fig. 6E) while the number of attached thrombocytes appeared to be slightly increased in the *f2*^Δ14/Δ14^ larvae, but only at 6 dpf (Fig. 6F). Overall these data reveal intact primary hemostasis but a loss of secondary hemostasis. Despite this, erythrocyte staining with o-dianisidine revealed no overt signs of hemorrhage in 1-week old *f2*^+/+^ or *f2*^Δ14/Δ14^ larvae (data not shown).

### An intact kringle 1 domain is important for normal prothrombin levels

**Figure 7:**
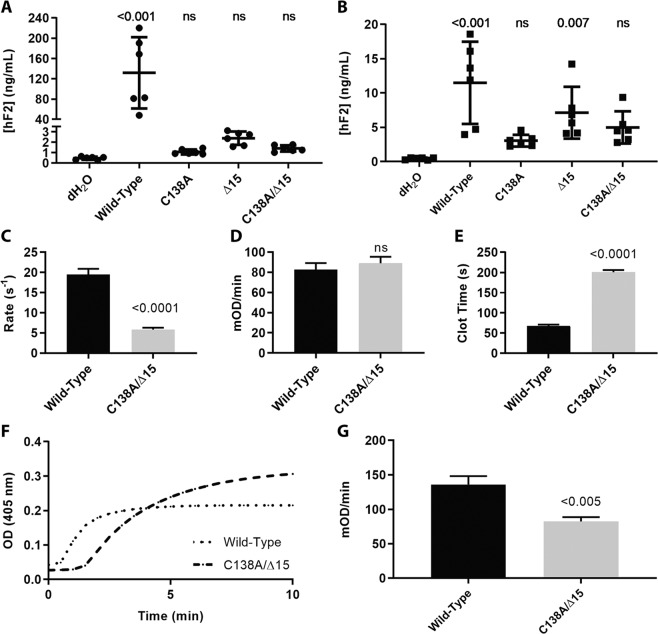
Expression and activation of prothrombin variants and resulting activity of thrombin upon activation. (**A**) Measurement of prothrombin in the cell media by ELISA demonstrated that expression of prothrombin variants in HEK293T cells resulted in reduced secretion levels that were indistinguishable from control and (**B**) corresponding measurement of prothrombin in the cell lysate demonstrated decreased biosynthesis. (**C**) Rates of prothrombin activation by prothrombinase measured using DAPA. Once prothrombin variants were fully activated by prothrombinase, their ability to cleave synthetic substrate S-2238 (**D**) or fibrinogen (**E**) were monitored, with significant differences in the latter, but not the former. For both S-2238 and fibrinogen, the rate was calculated during the initial reaction phase when the substrate is non-limiting and the conditions are presumed to be steady-state. Rate was then normalized to enzyme concentration. (**F**) Clotting profile between wild-type and C138A/Δ15 demonstrated delayed clot initiation and altered turbidity. (**G**) The rates of clot formation were determined from the clotting profile and reduced in the mutant.

To interrogate how partial loss of the kringle 1 domain alters prothrombin function, a Δ15 version of human prothrombin was generated, corresponding to the zebrafish deletion. As this deletion removes C114 and results in a free-thiol at C138, two additional prothrombin variants were generated (C138A and C138A/Δ15). Transient expression in HEK293T cells led to wild-type secretion >100 ng/mL while all mutants were <3 ng/mL and not significantly different from the non-transfected control (Fig. 7A). Furthermore, measurement of prothrombin in the cell lysate revealed >10 ng/mL for wild-type expression while two of the three mutants (C138A and C138A/Δ15) were <5 ng/mL and not significantly different from control. The remaining mutant, Δ15, was significantly different than control but on average produced less protein than the wild-type product (Fig. 7B). Overall, this suggests that the loss of these residues within kringle 1 impaired biosynthesis or stability and led to a significant reduction in overall secretion.

### The kringle 1 mutant has impaired endogenous activity despite an intact protease domain

The C138A/Δ15 prothrombin was expressed from a stably transfected HEK293 cell line, purified, and its activation by prothrombinase was quantified. The quantum yield of fluorescence signal change observed upon thrombin-DAPA complex formation was comparable between wild-type (3262 ± 347 RFU/µM) and C138A/Δ15 (3354 ± 180 RFU/µM), consistent with the protease domain remaining intact. However, the initial rate of activation, defined as the linear phase of steady state kinetics during the first 15–20% of the reaction, was 3.3-fold faster for wild-type (19.5 ± 1.4 s^−1^) compared with C138A/Δ15 (5.8 ± 0.5 s^−1^) (Fig. 7C).

The functionality of thrombin generated from the mutant prothrombin variant was then investigated. The amidolytic activity of thrombin towards S-2238 was similar between wild-type (82.7 ± 6.5 mOD/min) and C138A/Δ15 (89.0 ± 6.4 mOD/min) (Fig. 7D). However, C138A/Δ15-thrombin had a significantly prolonged clot time (200.6 ± 5.3 s) compared to wild-type (67.7 ± 3.1 s) (Fig. 7E), mostly due to delayed clot initiation and 1.5-fold higher total turbidity change (Fig. 7F) compared with wild-type. The rate of clot formation was also slower (82.4 ± 6.3 mOD/min) compared with the wild-type (135.9 ± 12.3 mOD/min) (Fig. 7G). These data suggest that thrombin derived from C138A/Δ15 prothrombin has normal catalytic properties towards small peptidyl substrates, but impaired activity towards macromolecular substrates like fibrinogen.

## Discussion

The zebrafish model has been developed as a useful tool for understanding coagulation, especially during early development. In contrast to mammals, in which a number of coagulation factors are necessary for embryonic and/or neonatal viability, zebrafish are able to survive the loss of many aspects of the canonical cascade at least until early adulthood. Loss of antithrombin, fibrinogen, FV, and FX are all compatible with development to adulthood^22,26–28,37^. Targeting of *f10* in fish results in the absence of larval hemostasis, but is accompanied by extended survival^28^. These data suggest that there could be residual thrombin activity present. Previous studies have utilized transient knockdown and chemical inhibition to study the loss of prothrombin in zebrafish^29,38–40^, providing valuable insight into the conserved function of prothrombin and suggesting a potential role for the coagulation cascade in development. Unfortunately, these technologies are susceptible to toxicity and off target effects. Additionally, specific chemical inhibition targets the proteolytic activity of thrombin without addressing the potential for exosite mediated binding of other factors. We sought to leverage knockout technology to create a specific genetic knockout of *f2*. We produced a genomic deletion in exon 6 with the intent of creating a nonsense mutation. Unexpectedly, we found reduced expression of an alternatively spliced version of prothrombin lacking a conserved cysteine in the kringle 1 domain. *In vitro* biochemical studies demonstrated that this protein is synthesized and secreted at a low level, and the secreted protein has decreased activation and activity. *In vivo*, the Δ15 mutant results in the loss of the ability to form fibrin rich clots and lethality by 2 months of age due to spontaneous hemorrhage. However, thrombocyte attachment was unaffected, consistent with the dogma that platelet adherence is mediated primarily via von Willebrand factor and collagen binding^41–43^. Overall, our data suggest that this is likely a severe hypomorph, if not functionally a complete null. Given that *f2*^Δ14/Δ14^ phenocopies the previously described *f10* knockout, it appears that any residual thrombin activity is likely to be physiologically negligible.

Surprisingly, no obvious developmental defects were observed, including grossly normal vascular development, with survival through early adulthood. Previous work using antisense technology has demonstrated a variety of developmental malformations including hemorrhage, and circulatory, brain, and tailbud malformations^38^. These defects were not observed across multiple clutches in our knockout. This inconsistency could be due generally to off target effects of antisense knockdown or genetic compensation that can occur in response to genomic editing^44^. Conversely, the discrepancy may be a result of the fibrinogen-independent residual activity of the mutant thrombin, as detected in the amidolytic assay. Notably, the *victoria* mutant, which mapped close to *f2* and is likely a mutant allele, similarly affects induced thrombus formation without a described developmental defect^45^, consistent with our data.

Kringle 1 is an understudied domain of prothrombin, but it is thought to interact directly with FVa and regulate prothrombin activation^11^. For the first time, we demonstrate that a defect in the kringle 1 domain may lead to decreased levels of prothrombin in circulation as well as decreased thrombin generation, thus contributing to the observed coagulopathy. The impairment of overall thrombin activity occurs at multiple levels. First, presumed inefficiency of the cryptic splice site in mRNA maturation leads to a roughly 45% reduction in transcript levels due to predicted nonsense mediated decay of the mutant transcript making use of the canonical splice site. Notably, splicing to the cryptic site is not seen in wild-type fish and a cryptic AG splice acceptor is not present in the human transcript. Second, when introduced into human prothrombin, the deletion resulted in a substantial decrease in secretion. It is possible that a large amount of the mutant prothrombin misfolds, aggregates, and is degraded intracellularly. Additionally, low secretion may be indicative of a more involved role of the kringle 1 domain in regulating prothrombin secretion as has been previously suggested^46^. The C138A/Δ15 prothrombin derivative (controlling for the unpaired C138 resulting from Δ15) showed a 3-fold reduction in its rate of activation by prothrombinase when compared with the wild-type prothrombin. In addition, thrombin generated from the C138A/Δ15 variant had a decreased procoagulant function (*i.e*. macromolecular substrates) while maintaining a functional active site as demonstrated by the unaltered S-2238 hydrolysis (*i.e*. small synthetic substrates) when compared with wild-type thrombin. Although similar thrombin functionality towards S-2238 was expected since the mutations did not involve the protease domain of prothrombin (i.e. thrombin), the delineation of substrate specificity towards fibrinogen was unexpected. This variation in substrate specificity could be explained by incomplete cleavage leading to an accumulation of a meizothrombin intermediate^47^. However, we did not observe the associated increase in DAPA-thrombin fluorescence predicted by increased meizothrombin presence. Conversely, our findings propose the potential role of F1.2 in the substrate recognition and specificity of thrombin, whereby the disruption of the kringle 1 domain leads to reduced fibrinogen recognition and/or cleavage by the F1.2:thrombin complex. Therefore, our data suggest that F1.2 is more than just a peptide fragment that results in product-inhibition of thrombin^48^. Overall, the compounding reductions in transcription, secretion, activation, and activity show that this genomic deletion results in a severe hypomorph. At the same time our data propose a role for the kringle 1 domain in the activation kinetics and subsequent substrate recognition of thrombin.

The ability of zebrafish to survive severe hemostatic imbalance may be due to a combination of influences, including the absence of birthing trauma, limited hemostatic challenges in an aqueous laboratory environment, a relatively low systolic blood pressure, and possible species-specific genetic differences. Nevertheless, the phenotypes of all zebrafish hemostatic mutants eventually converge with their mammalian counterparts in adulthood. Overall, this study further demonstrates the conservation of the coagulation cascade in fish while leveraging unique physiologic differences to build our understanding of the complex biological functions of thrombin. Furthermore, understanding how fish tolerate such a severe bleeding diathesis could provide new insights into managing patients with congenital bleeding disorders or acquired hemorrhage, with future studies leveraging the model system to develop new diagnostic markers and therapies.

## Methods

### Animal care

Zebrafish were maintained according to protocols approved by the University of Michigan Animal Care and Use Committee. All wild-type fish were a hybrid line generated by crossing AB and TL fish acquired from the Zebrafish International Resource Center. A hybrid line was selected to minimize strain specific effects and for improved reproductive success^49^. *Tg(cd41:egfp)* fish were used for tracking of fluorescently labeled thrombocytes^50^. Tris-buffered tricaine methanosulfate (Western Chemical) was used for anesthesia during all live animal experimental manipulation and for euthanasia.

### Sequence analysis

Prothrombin protein sequences (NP_000497.1, NP_034298.1, NP_989936.1, NP_998555.1, NP_001015797.1) were downloaded from the National Center for Biotechnology Information RefSeq database^51^. Homology predicted propeptides were removed and the prothrombin numbering scheme^52^ was used relative to the human prothrombin sequence unless otherwise noted. Zebrafish sequence was modified to include an incompletely annotated exon. Sequences were aligned using MUSCLE^53^ and shaded using the Boxshade server (https://embnet.vital-it.ch/software/BOX_form.html).

### Targeted mutagenesis using TALENs

The *f2* coding sequence was identified in the zebrafish reference genome (Gene ID: 325881)^54^. Factoring in technical design limitations and predicted efficacy, an optimal target site 5’ to the protease domain was selected in exon 6 for mutagenesis. TALEN constructs were created using fast ligation-based solid-phase high-throughput (FLASH) assembly targeted to exon 6 of the zebrafish genomic *f2* locus and validated as described^55,56^. TALEN mRNA was transcribed from plasmids and injected into single cell embryos. The resulting chimeric founders were raised, outcrossed, and offspring screened for deleterious mutations. A single founder heterozygous for a 14 base pair deletion (*f2*^Δ14^) in the coding sequence was identified and crossed to wild-type fish to establish the mutant line.

### Genotyping of mutant offspring

Whole embryos or adult fin biopsies were lysed in buffer (10 mM Tris-HCl, pH 8.0, 2 mM EDTA, 2% Triton X-100, 100 µg/mL proteinase K) for 2 hours at 55 °C followed by incubation at 95 °C for 5 minutes. One microliter of the resulting solution was used as template for gene specific polymerase chain reaction (Table S1) and analyzed by gel electrophoresis.

### Histochemical analysis

For *in situ* hybridization, DIG-labeled riboprobes (DIG RNA-labeling kit, Roche) were synthesized using 2-day old wild type embryonic cDNA and gene specific primers with T7 overhangs (Table S1). Embryos were fixed overnight at 4 °C in 4% paraformaldehyde in phosphate buffered saline prior to dehydration. Permeabilization and staining were performed as described^57^. Stained samples were evaluated by phenotype prior to genotyping.

For hematoxylin and eosin staining, juvenile zebrafish (30–89 days) were fixed overnight in 4% phosphate buffered paraformaldehyde and embedded in paraffin. Sagittal sections (3 µm) were collected every 50 µm and stained.

### Single molecule real-time sequencing of RNA

Three *f2*^+/Δ14^ larvae were homogenized at 5 dpf in lysis buffer using a 21-gauge syringe. Total RNA was purified from the lysate using the PureLink RNA Mini Kit (Life Technologies) followed by DNAse I treatment (Invitrogen) and cDNA synthesis using the Superscript III First Strand cDNA kit (Invitrogen). Primers (Table S1) were used to amplify a 918 base pair (bp) region surrounding the predicted deletion. This region included several non-deleterious single nucleotide polymorphisms (SNPs) in downstream exons known to be allelic with either the wild-type or mutant alleles. Purified products from the 3 samples were pooled and sent to the University of Michigan Sequencing Core for library preparation and single molecule real-time high throughput sequencing (SMRT, Pacific Biosciences). Circular consensus reads with at least 5x coverage were filtered for full-length single inserts. After filtering for quality, the resulting 3826 reads were sorted by haplotype and splice variation.

### Quantitative real-time PCR

Total RNA was extracted from 7 days post fertilization larvae (dpf) using the RNeasy Mini Plus kit (Qiagen) and transcribed using oligo(dT)_12–18_ primer and Superscript III (Invitrogen). Three pools of three whole larvae were used per genotype. The resulting cDNA was used as template for qPCR (StepOnePlus, Applied Biosystems) using gene specific PrimeTime^©^ probe-based qPCR Assays (Integrated DNA Technologies) (Table S1). The expression level of *f2* was normalized to the *actb2* gene and significance analyzed using the double delta Ct method as described^58^. Transcript flanking the deletion was amplified using allele unbiased primers and separated by capillary gel electrophoresis (Table S1). Molar ratios were calculated from standardized relative band intensities using QIAxcel ScreenGel software (Qiagen).

### Laser-induced endothelial injury

Anesthetized zebrafish larvae were embedded in 0.8% low melting point agarose and oriented in the sagittal position on a glass coverslip. Larvae were then positioned under an Olympus IX73 inverted microscope with an attached pulsed nitrogen dye laser (Micropoint, Andor Technology). For venous injury, ninety-nine laser pulses were administered to the luminal surface of the endothelium on the ventral side of the posterior cardinal vein (PCV) 5 somites posterior to the anal pore of 3 dpf larvae as previously described^59^. For arterial injury, pulses were administered to the endothelial surface of the dorsal aorta 3 somites posterior to the anal pore of 5 dpf larvae^59^. Following injury, time to occlusion and/or time to thrombocyte attachment were monitored for 2 minutes. Larvae were manually extracted from agarose for genotyping.

### Chemical treatment

Dabigatran etexilate, apixaban, and rivaroxaban were dissolved in DMSO and diluted in embryo water to final concentrations of 50, 100, and 250 µM, respectively. At 5 dpf larvae were treated for 24 hours prior to laser-mediated endothelial injury on day 6. For ε-aminocaproic acid (Sigma) treatment, zebrafish embryos were incubated in 100 mM at 1 dpf and the time to occlusion was evaluated at 3 dpf following laser-induced endothelial injury.

### O-dianisidine staining

Anesthetized larvae were incubated in the dark at 4 dpf for 30 minutes in o-dianisidine staining solution as previously described^60,61^. Larvae were subsequently fixed overnight at 4 °C in 4% paraformaldehyde, and pigment was removed using bleaching solution (1% KOH, 3% H_2_O_2_).

### Human prothrombin expression vector construction and injection

Plasmid pF2-bio-His was obtained from Addgene (plasmid #52179) and human *F2* cDNA amplified using primers containing vector sequence homology (Table S1). pcDNA3.1 was digested with BstBI and KpnI, followed by gel purification of the linearized backbone. The NEBuilder kit was used to fuse the *F2* cDNA with linearized vector and transformed into 10beta competent cells (NEB). Site directed mutagenesis was used for missense mutation introduction^62^ and NEBuilder end homology joining was used to create internal deletions. Replacement of residues 100 to 115 of human F2 with a glutamic acid resulted in a homologous 15 amino acid deletion in human prothrombin (Δ15). As the deletion includes the cysteine residue at position 114, the effect of introducing a free-cysteine at position 138 due to the loss of its binding partner was investigated by generating two additional vectors that included an alanine substitution without (C138A) or with the 15-residue deletion (C138A/Δ15). For *in vivo* rescue assays, plasmid DNA (90 pg) in 0.1 M KCl was injected into one-cell stage embryos generated from heterozygous *f2*^Δ14^ incrosses using pulled glass capillary needles.

### Human prothrombin protein expression and isolation

*F2* mutant vectors were transfected into human embryonic kidney 293 T (HEK293T) cells in 6-well plates using Lipofectamine 3000 (ThermoFisher Scientific; L3000008) according to manufacturer’s instructions. The media and cells were collected after 3 days. The cells were washed with DPBS and lysed with 50 µL RIPA buffer on ice for 15 minutes. Prothrombin concentration in the expression media and cell lysate were quantified by ELISA using a matched-pair antibody set (Affinity Biologicals Inc., Ancaster, Canada), according to manufacturer’s instructions.

To characterize the functional characteristics of the prothrombin derivative, the plasmid containing the cDNA for prothrombin C138A/Δ15 was transfected and selected in HEK293 cells to generate a stable cell line. Using sustained rolling incubation at 37 °C, expression was induced in D-MEM supplemented with vitamin K_1_, and collected every 2–3 days^63^. The media were then centrifuged at 2,000 × g to remove cell debris, and the supernatant was filtered through a 0.22 µm filter (Millipore). The media was loaded onto tandem XAD_2_ and Q-Sepharose fast flow columns pre-equilibrated with 0.02 M Tris, 0.15 M NaCl, pH 7.4 (TBS) and eluted with 0.02 M Tris, 0.5 M NaCl, pH 7.4 as described previously^64^.

### Prothrombin activation and activity

Prothrombin activation was quantified as described previously^64^. Briefly, prothrombin (0.1 µM) was incubated with PCPS vesicles (50 µM; 75% phosphatidylcholine, 25% phosphatidylserine), FVa (20 nM), CaCl_2_ (5 mM), and dansylarginine *N*-(3-ethyl-1,5-pentanediyl)amide (1 µM, DAPA) in 0.02 M HEPES, 0.15 M NaCl, pH 7.4 with 0.01% Tween80 (HBST). The reactions were initiated by the addition of FXa (70 pM) and activation was monitored for fluorescence change at 1 minute intervals using a SpectraMax M2 plate reader (Molecular Devices). Excitation and emission spectra were 280 nm and 540 nm, respectively, with an emission cutoff filter set at 530 nm. The quantum yield of the thrombin-DAPA complex was determined by plotting the total signal change observed with respect to known concentrations of the thrombin-DAPA complex^12^.

To determine the resulting thrombin activity, prothrombin was activated to thrombin by prothrombinase as described above. After a 30-minute incubation, complete prothrombin activation was verified by SDS-PAGE (not shown). The resulting thrombin (5 nM) was simultaneously added to either a thrombin-specific substrate S-2238 (400 µM; DiaPharma, West Chester, OH) or fibrinogen (3 µM), all in the presence of 5 mM CaCl_2_ in HBST. Both reactions were monitored at 405 nm using SpectraMax M2 plate reader (Molecular Devices) to quantify the amidolytic (S-2238) or clotting (fibrinogen) activity of thrombin.

### Statistical analysis

The occlusion data were analyzed using Mann-Whitney *U* or two-tailed Student *t* tests. Survival was evaluated by log-rank (Mantel-Cox) testing. Significance testing, graphs, and survival curves were made using GraphPad Prism (Graphpad Software, La Jolla, California). P-values (p < 0.05 or p < 0.0001) were used to evaluate statistical significance.

All data generated or analyzed during this study are included in this published article (and its Supplementary Information Files).

## Supplementary Information

Supplementary Information.
