# Supplementary material for: Disruption of the kringle 1 domain of prothrombin leads to late onset mortality in zebrafish
Source: Sci Rep. 2020 Mar 4;10:4049. doi: 10.1038/s41598-020-60840-7 (PMC7055286; doi:10.1038/s41598-020-60840-7)
Supplement: Supplementary file 1 — Supplementary Information. [file 41598_2020_60840_MOESM1_ESM.pdf]

Table S1: Oligonucleotide sequences used in methods

Primer Description	Primer Sequence
f2 qPCR Forward	GAGGAAGCTCGAGAAGTGTTT
f2 qPCR Reverse	CCCTCTGTAGTCGCACATATTC
f2 qPCR Probe	FAM/TGGACAGGG/ZEN/TTGTTCCTTCACAGC/IABkFQ
actb2 qPCR Forward	ATGAAGATCCTGACCGAGAGA
actb2 qPCR Reverse	TCAAAGTCAAGGGCCACATAG
actb2 qPCR Probe	FAM/ACCACCACA/ZEN/GCTGAGAGGGAAATT/IABkFQ
f2 delta Flanking Forward	GAGGAAGCTCGAGAAGTGTTT
f2 delta Flanking Reverse	AAAACACCAAGGGCCATCTTT
f2 genotyping Forward	TGCCTTTAGTGATGTTCCTCTG
f2 genotyping Reverse	CTGACAGTCGGGTCTCTGGT
F2 SMRT sequencing Forward	AGGCTGTGAAGGAACAACC
F2 SMRT sequencing Reverse	CTGATCAGACTGGCTCCAC
human F2 with vector homology Forward	TGGCTAGTTAAGCTTGGTACATGGCTCATGTGCGGGGCC
human F2 with vector homology Reverse	TTAGGGATAGGCTTACCTTCTCCGAACTGGTCGATCAC
human F2 Δ45 mutagenesis Forward	CGAGATCGAGAACCCCGATAGCAGCACCACC
human F2 Δ45 mutagenesis Reverse	CGGGGTTCTCGATCTCGGGCTTGTGGGGGT
human F2 C138A mutagenesis Forward	GAGACAGGAAGCATCGATCCCCGTCTGCGGCCAGGATCAGG
human F2 C138A mutagenesis Reverse	AGACGGGGATCGATGCTTCCTGTCTCCGCACGGTGGGGTCGG
Antisense Riboprobe + T7 Forward	TAATACGACTCACTATAGGGGTGATGATGACCGAGGTGGA
Antisense Riboprobe Reverse	CCGACTGTCAGGAGGGAGAC
